# Comparative study on mineral dissemination characteristics of phosphate ores by X-ray micro computed tomography and BGRIMM Process Mineralogy Analysis

**DOI:** 10.1038/s41598-022-24671-y

**Published:** 2022-12-07

**Authors:** Jun Yang, Yueqin Qiu

**Affiliations:** 1 School of Mining, Guizhou University, Guiyang, 550025 China; 2 National & Local Joint Laboratory of Engineering for Effective Utilization of Regional Mineral Resources From Karst Areas, Guiyang, 550025 China; 3 Guizhou Key Laboratory of Comprehensive Utilization of Non-Metallic Mineral Resources, Guiyang, 550025 China

**Keywords:** Mineralogy, Solid Earth sciences

## Abstract

Because the 2D (two-dimensional) characterization analysis of mineral dissemination characteristics requires complex sample preparation, destroys the sample structure, and produces stereological errors, a new method for analyzing mineral dissemination characteristics in the ore in situ, non-destructively and stereoscopically, is required. The research object in this paper is a medium–low grade calcareous-magnesium phosphate ore in Guizhou, and XMT (X-ray microtomography) and BPMA (BGRIMM Process Mineralogy Analysis) are used to conduct a comparative study of 3D (three-dimensional) and 2D analysis of mineral dissemination characteristics. The results of grain size analysis show that fluorapatite and gangue minerals belong to equal-grain dissemination, with very little fine particle content. The results of 2D analysis are finer than those of 3D analysis, but 3D non-destructive analysis produces more accurate results. In addition to particle size analysis, the binding relationship between minerals analysis results show that, when compared to the 2D distribution of minerals, 3D visualization can more intuitively and stereoscopically observe the distribution of minerals inside the ore and the intergrowth relationship between minerals. Through comparative study, it can be seen that the application of 3D visualization has developed a new method for the study of mineral dissemination characteristics, which makes up for the shortcomings of 2D analysis.

## Introduction

The main source of phosphorus is phosphate rock, which is widely used in medicine, industry, food, and other fields^1,2^. According to mineralization, phosphate rock can be classified as igneous, sedimentary, or biological. Sedimentary rocks account for approximately 75% of phosphate rock resources. With the depletion of rich mineral resources, phosphate rock resources are becoming increasingly important as a non-renewable resource. Flotation is the most effective separation method in the phosphate rock processing process, and it is used to process more than 60% of the world's phosphate rocks^3^. However, the useful minerals in medium–low grade phosphate rocks are mostly fine-grained collophane, and the properties of the main gangue minerals, dolomite and calcite, are similar to those of collophane, making flotation separation of medium–low grade phosphate rocks difficult^4^. As a result, an important research topic is the process mineralogy analysis of ore structure, mineral dissemination characteristics, and dissociation characteristics prior to flotation separation. Among them, the dissemination characteristics of minerals refer to the particle size, shape and the combination relationship between minerals in the ore. It is a comprehensive concept related to the spatial morphology of minerals. The analysis results show that the mineral morphology has an important effect on the beneficiation process, especially on the crushing and grinding. According to studies, the ore crushing process consumes the most energy^5,6^, and the appropriate crushing process can not only reduce energy consumption but also promote mineral dissociation and improve flotation index. To increase flotation efficiency and reduce energy consumption, it is crucial to analyse the properties of mineral dissemination.

The research methods of process mineralogy of ores have changed from the original optical microscope to the commonly used scanning electron microscope-based mineral liberation analysis (MLA) or the quantitative evaluation of minerals by scanning electron microscopy (QEMSCAN)^7,8^. However, these techniques necessitate slicing the ore sample in order to expose the 2D surface and observe the internal structure of the sample^9^. As a result, these technologies are destructive and intrusive, and they can only obtain 2D information. As a result, stereological correction is required for particle size and mineral dissociation analysis, which computer tomography (CT) can overcome. The technique is non-destructive and can reveal the internal structure of an object based on X-ray densities. The technology was first used in the medical field, but it has also been used in other fields since the 1990s, first in petroleum geology^10,11^, then in process mineralogy^12^ and process technology^13,14^.

In contrast to medical CT, CT in mineralogy requires more light source energy and higher resolution, and the most commonly used CT is XMT. The use of XMT in mineralogy is a relatively new but rapidly expanding application area^15^. Many researchers have discovered that the technology has clear advantages in mineral analysis when the particle size is coarse and the sample is metal ore. This is due to the fact that the attenuation values of useful minerals and gangue minerals in metal ores differ significantly, and the image is easy to process due to the high contrast. By obtaining 3D information of mineral size, shape, and location in two sulfide ores, Pratama Istiadi Guntoro et al.^16,17^ developed a 3D liberation model. Takao Ueda^18^ performed 3D characterization of the self-made copper-silicate two-phase ore, obtained the volume proportion of copper in different particles and the surface exposure data, and studied the stereological deviation of binary particles. Wang et al.^19^ conducted an embodied study on the perimeter-based surface area estimation of exposed particles using copper ore. However, few studies on the application of mineralogy in non-metallic minerals have been conducted because the attenuation values of useful minerals and gangue minerals in non-metallic minerals are close, and phase segmentation in image processing is difficult. Some researchers use XMT to study coal selectivity by analyzing the density composition and particle characteristics of coal^20–22^. However, for the application research of phosphate rock, Puvvada et al.^23^ studied the process mineralogy of phosphate ore with + 1 mm particle size, proving the feasibility of the application of this technology in the field of process mineralogy of phosphate rock. In the process of flotation separation of phosphate rock, it is still of great guiding significance to study the process mineralogy of ore particles with particle size of − 1 mm or finer. The purpose of this paper is to use XMT to analyze the mineral dissemination characteristics of fine-grained calcium-magnesium phosphate ore and compare it with the traditional 2D characterization. A new method of 3D visual dissemination characteristics is introduced to reveal the structure of medium–low grade calcium-magnesium phosphate ore, which makes up for the deficiency of 2D characterization.

## Materials and methods

### Ore sample

The ore samples were taken from medium–low grade calcium magnesium phosphate ore in Guizhou's phosphate rock area and crushed to − 1 mm. Fluorapatite, dolomite and quartz pure minerals are selected from the high-grade phosphate rock in the phosphate rock area. XRF (X-ray fluorescence spectroscopy) was used to analyze the chemical composition of raw ore and three pure minerals. The instrument model was Panalytical Zetium from the Netherlands. The test method was tableting method and the test mode was oxide mode. XRD (X-ray powder diffraction) was used to analyze the mineral composition of the raw ore and three pure minerals. The instrument model was Bruker D8 Advance, Germany. The scanning speed of the raw ore was 2°/min, and the scanning speed of the pure mineral was 5°/min. The test targets were all Cu targets, and the scanning angle range was 5–90°. The content of mineral components in raw ore samples can be obtained by automatic analysis system of process mineralogy.

### XMT analysis

#### XMT scan

The raw ore samples of + 250 μm and − 250 μm were collected by using Nikon XTH 225 scanner for XMT raw data collection. According to Ralf Ditscherlein et al.^24^ the samples with sufficient number of ore particles and representativeness were selected. The samples of + 250 μm and − 250 μm were respectively scaled to 10 g and 0.35 g, respectively. Paraffin was used as a fixative to prevent the movement of ore particles in the scanning process from causing errors. PVC plastic tubes with diameters of 10 mm and 6 mm were used as containers for storing particles, and three pure minerals were embedded at the top of the particle filling column as standard samples. The detector of the CT scanning instrument is composed of 2000 × 2000 detector matrices, which can provide a resolution of more than 1 μm for two samples. The scanning parameters are shown in Table 1.

**Table 1:** Scanning conditions of XMT.

Parameter	Numerical value	
	+ 250 μm	− 250 μm
Voltage	120 kV	120 kV
Current	160 µA	160 µA
Light filter	0.1 mm copper sheet	0.1 mm copper sheet
Exposure time	0.708 s	0.708 s
Projection torus times	2000	2000
Resolution	28.29 μm	3.65 μm

**Figure 1:**
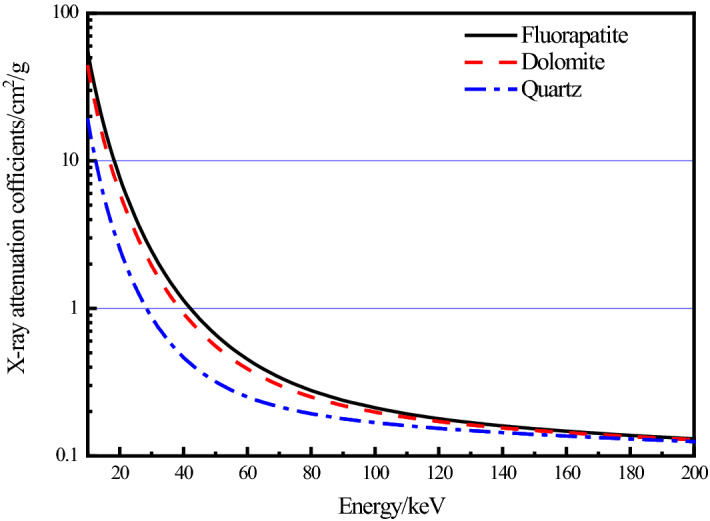
Theoretical X-ray attenuation values of three minerals at different energy levels.

Using the program XMuDat^25,26^ (Photon Attenuation Data on PC Version 1.0.1 of August 1998-Summary), the linear attenuation coefficients of fluorapatite, dolomite, and quartz at various X-ray energy levels are calculated, as shown in Fig. 1. Although the X-ray light source in the CT scanning process typically uses a multi-color light source, which is different from the monochromatic light beam provided by the software XMuDat, the functional relationship between their energy and the linear attenuation coefficient is the same. This is because the software XMuDat bases its linear relationship between the X-ray mass attenuation coefficient and energy on a monochromatic light source. Fluorapatite has the highest theoretical attenuation value in the group, and it and quartz have different attenuation values that may be easily distinguished. Fluorapatite and dolomite have similar attenuation values, which could make segmentation challenging.

#### 3D reconstruction and image preprocessing

**Figure 2:**
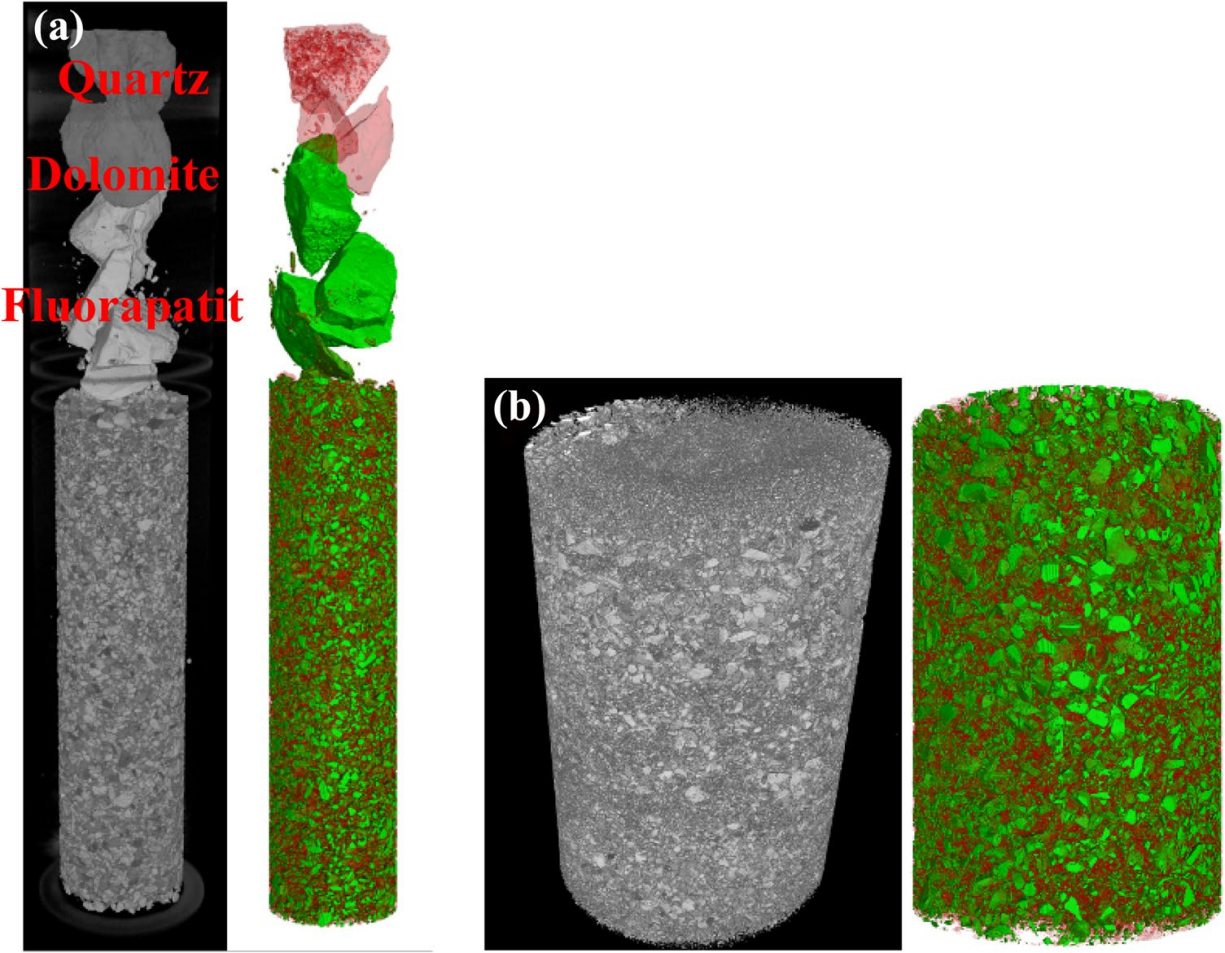
3D reconstruction of the (**a**) + 250 µm and (**b**) − 250 µm fractions.

**Figure 3:**
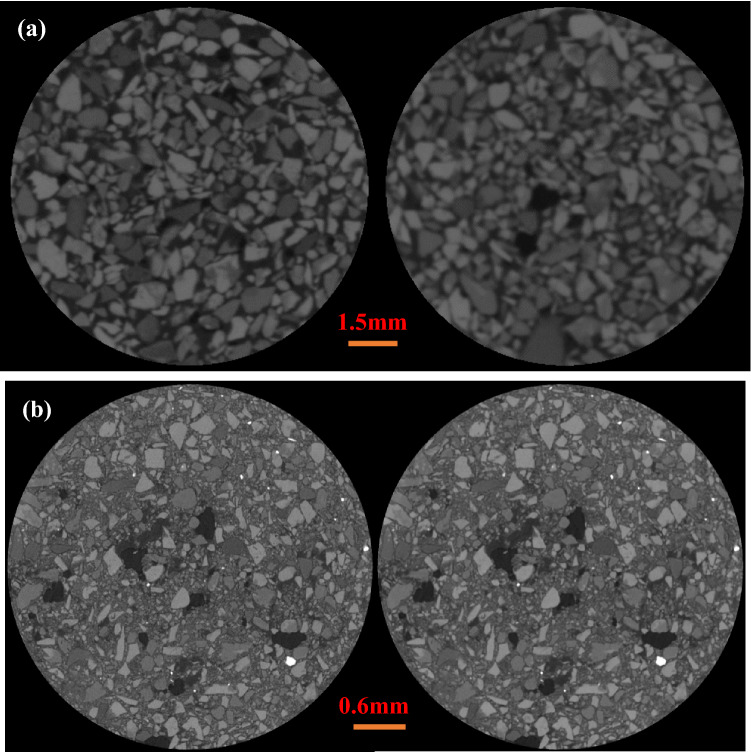
(**a**) + 250 µm and (**b**) − 250 µm fractions of original and processed images; left image before processing, right image after processing.

The final voxel resolution of the scanning is 28.29 µm for a + 250 µm grain size, and 4296 slices are acquired using the procedure described above for selecting the scanning parameter settings. 5382 slices are scanned and acquired, with some samples having voxel resolutions of 3.65 µm at grain sizes of less than 250 µm. The original picture data is divided into two groups. The original data of two grain sizes is recreated using the filtered back projection algorithm, as illustrated in Fig. 2. Fluorapatite, dolomite, and quartz, which are all pure minerals, were positioned at the top of the sample column in the image to adjust for beam hardening. The 2D slices of the two samples were denoised by adaptive Gaussian filtering and median filtering, respectively, to enhance the image quality and increase the accuracy of the future processing technique. Figure 3 displays both the denoised image and the original data image.

**Figure 4:**
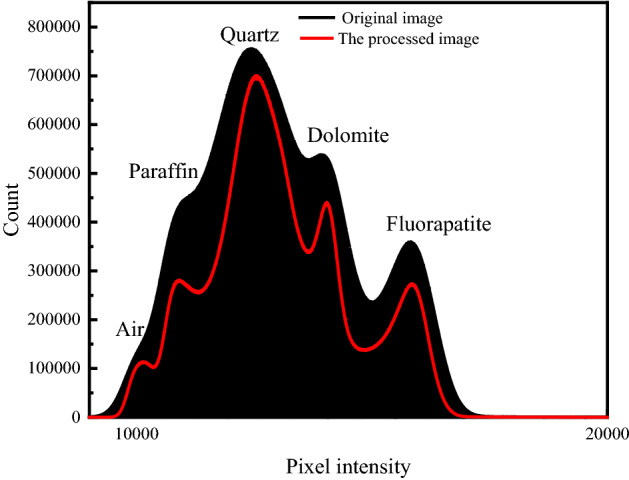
Histogram comparison before and after image processing.

The gray value of fluorapatite is the largest, the pixel intensity is the largest, followed by dolomite, and the lowest is quartz when the attenuation coefficient analyses of the three minerals are combined. Changes in pixel strength before and after processing, as shown in Fig. 4. Figure 4 shows that the average pixel intensity difference of the three minerals is small before the pretreatment, and that the pixel intensity difference of the three minerals increases after the pretreatment. It is worth noting that the pixel intensity difference between dolomite and quartz is small before and after the pretreatment because the attenuation coefficient of the two minerals is small. As a result, the primary focus of this article is on the fluorapatite target mineral's subsequent image processing, while dolomite and quartz are considered gangue minerals.

#### Image segmentation of particles

**Figure 5:**
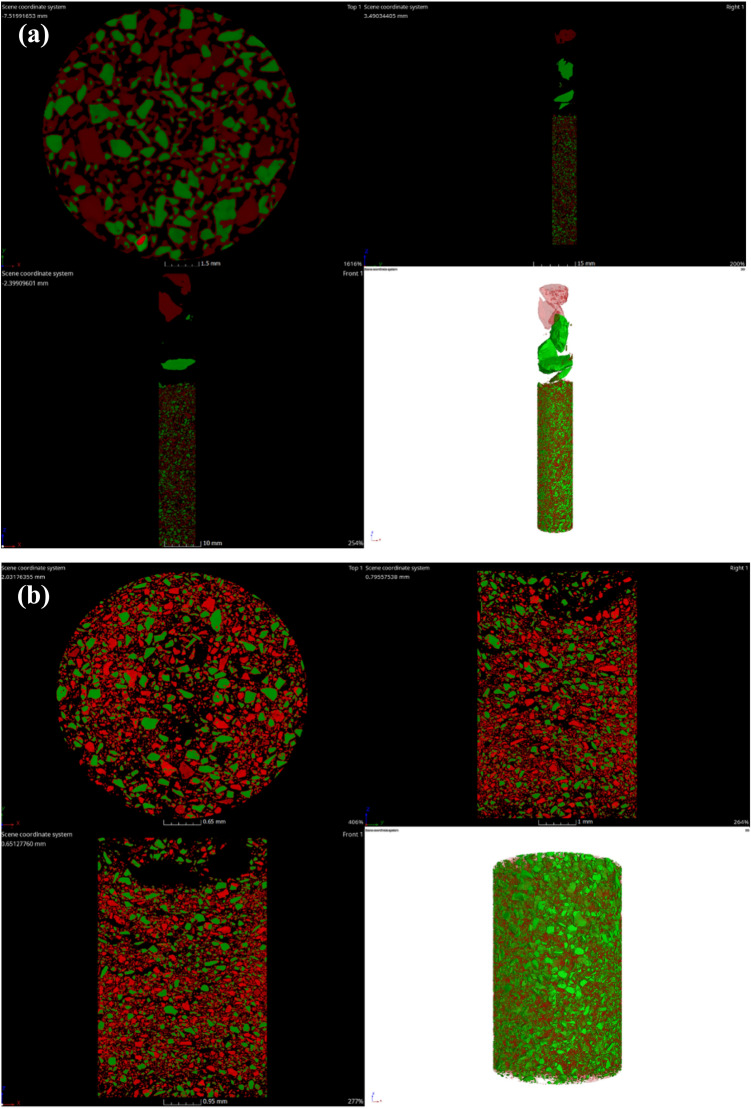
(**a**) + 250 µm and (**b**) − 250 µm fractional image segmentation results.

After the original image is denoised, the watershed algorithm is used to segment the mineral phases and pores of fluorapatite and other gangue minerals based on the gray value, in which green represents fluorapatite and red represents gangue minerals. In order to highlight the contrast between the two minerals, after the segmentation of the 2D slice, the color of gangue minerals in the reconstructed 3D model is operated to improve transparency, and the 3D segmentation image is obtained as shown in Fig. 5.

### BPMA analysis

Firstly, the Olympus CX21P microscope was used to observe and analyze the morphology of the three main minerals and the binding state between them. Then, 150 g of the raw ore particles in the whole particle size was divided and sampled, and an appropriate amount of the sample was prepared into a light sheet with a diameter of 30 mm by using epoxy resin, and then the light sheet was subjected to rough grinding, fine grinding, fine grinding and grinding and polishing treatment. The treated light sheet was naturally dried after ultrasonic decontamination treatment, and finally carbon spraying treatment was carried out to enable the sample to conduct electricity. The sample after carbon spraying was analyzed on the process mineralogy automatic test system BPMA^27^. It is an automatic electron microscope energy spectrum system that controls the automatic operation of scanning electron microscope and energy spectrum through API (Application Program Interface). During the test, the scanning electron microscope is continuously measured in the high vacuum state of the sample chamber. The measurement method is backscatter mode. The acceleration voltage is set to 20 kV, and the working distance is 15.11 mm. The magnification is adjusted according to the actual observation needs. Finally, the test is completed in the full particle measurement mode of the system.

## Result and discussion

### Analysis results of ore properties

**Figure 6:**
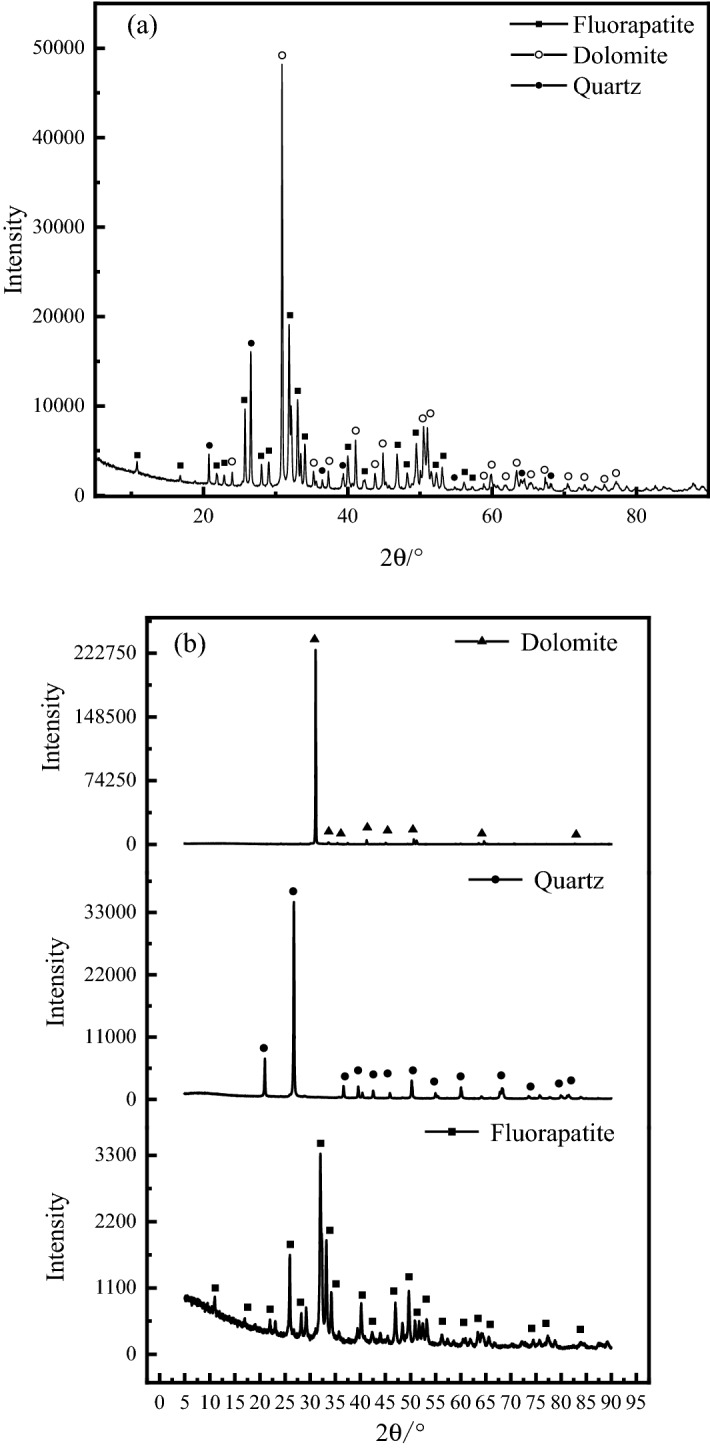
XRD analysis results of raw ore and pure mineral.

The results of mineral composition and chemical composition analysis of raw ore and three pure minerals are shown in Table 2 and Fig. 6, respectively. The mineral content analysis of raw ore is shown in Table 3.

**Table 2:** Chemical composition of raw ore and pure mineral samples.

Elemental composition	P_2_O_5_/%	CaO/%	MgO/%	SiO_2_/%	F/%	Al_2_O_3_/%	Fe_2_O_3_/%	SO_3_/%	Other/%
Raw ore	21.09	45.55	7.90	10.16	1.50	0.99	0.53	0.36	11.93
Fluorapatite	38.79	54.78	0.091	1.32	2.94	0.035	0.09	0.747	1.21
Dolomite		44.6	21.04	0.089		0.034	0.086		34.08
Quartz		0.042		99.54		0.093	0.23		0.034

**Table 3:** Mineral composition and relative content of the samples.

Name	Content /%	Name	Content/%
Fluorapatite	52.68	Sericite	0.32
Dolomite	36.75	Barite	0.07
Quartz	7.84	Magnetite	0.36
Pyroxene	0.87	Crooked feldspar	0.31
Calcite	0.41	Other	0.30
Hastingsite	0.09	Total	100

According to the results of mineral composition analysis and mineral composition content analysis of the original ore, it can be seen that the ore mainly contains three minerals of fluorapatite, dolomite and quartz, with contents of 52.68%, 36.75% and 7.84%, respectively. At the same time, according to the results of chemical composition analysis, it can be seen that the contents of P_2_O_5_ and MgO in the original ore are 21.09% and 7.90%, respectively, which belong to medium–low grade calcium magnesium phosphate ore. Through the analysis of mineral composition and chemical composition of fluorapatite, dolomite and quartz, the purity of the three minerals is above 90%, which meets the requirements.

### Comparative analysis of mineral distribution

**Figure 7:**
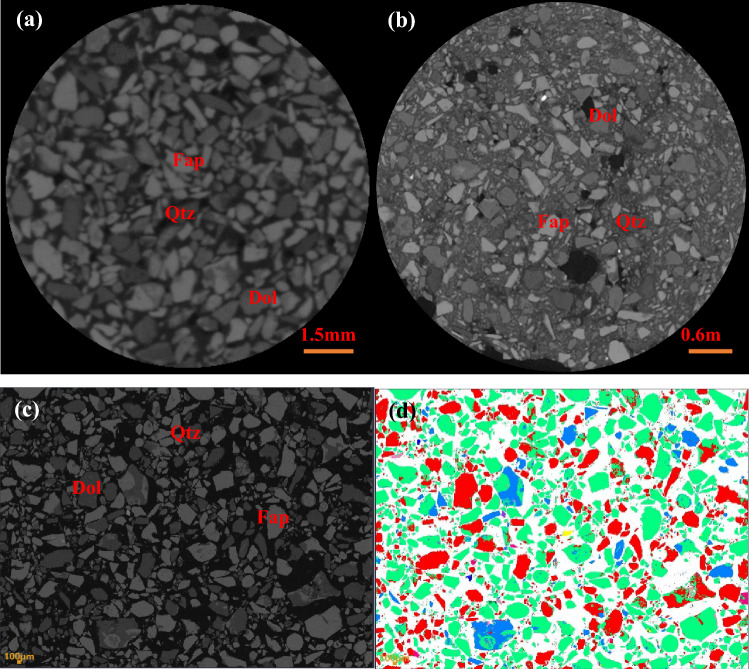
Comparison of results of three minerals under two scanning modes; (**a**) + 250 µm and (**b**) − 250 µm CT scan results, (**c**) is the SEM backscattering image of mineral distribution, and (**d**) is the SEM backscattering image after color rendering.

The findings of the mineral distribution image analysis performed by BPMA are displayed in Fig. 7. The red is dolomite, the blue is quartz, and the green is fluorapatite. Figure 7 shows three different types of pictures: 2D slice of XMT, SEM backscatter (BSE) images, and the results of mineral distribution.

Figure 7 demonstrates that the contrast between fluorapatite and quartz in an XMT image is better than the contrast between dolomite and quartz. The contrast of dolomite and quartz in the XMT image is better than that in the backscatter image taken with a scanning electron microscope. For the morphology of minerals, SEM-BSE images clearly observed the morphological characteristics of three main minerals than XMT 2D slice images.

### Comparative analysis of mineral particle size distribution

**Figure 8:**
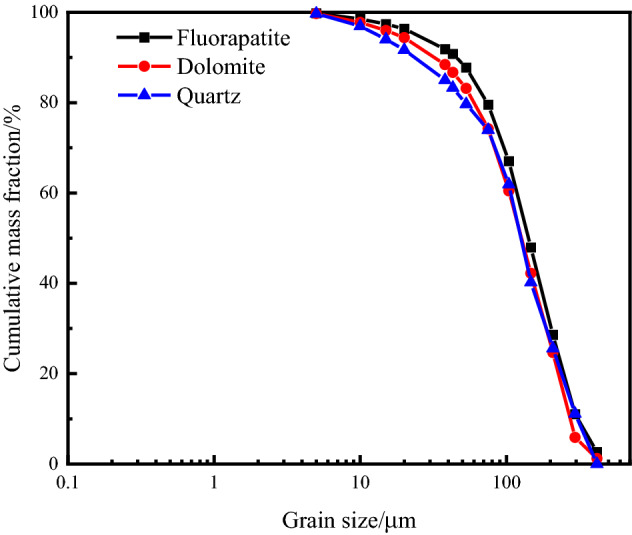
Semi-logarithmic cumulative particle size characteristic curve of main minerals.

The particle size of fluorapatite obtained from XMT analysis is represented by the equivalent spherical diameter, and the particle size analysis based on BPMA is represented by the elliptical short diameter of mineral particles. The results are shown in Table 4 and Fig. 8.

**Table 4:** Based on BPMA 2D chimeric grain size distribution.

Grain size/μm	Fluoraptite		Dolomite		Quartz	
	Content/%	Cumulative content/%	Content/%	Cumulative content/%	Content/%	Cumulative content/%
− 589 + 417	2.67	2.67	1.22	1.22	0	0
− 417 + 295	8.37	11.03	4.66	5.89	11.16	11.16
− 295 + 208	17.56	28.59	18.80	24.69	14.44	25.60
− 208 + 147	19.34	47.94	17.54	42.23	14.65	40.25
− 147 + 104	19.13	67.07	18.26	60.48	21.65	61.90
− 104 + 75	8.24	79.53	13.73	74.21	12.07	73.98
− 75 + 53	8.24	87.77	8.96	83.17	5.70	79.68
− 53 + 43	3.01	90.78	3.54	86.72	3.63	83.31
− 43 + 38	1.06	91.84	1.73	88.45	1.74	85.05
− 38 + 20	4.50	96.35	5.95	94.40	6.65	91.70
− 20 + 15	1.06	97.41	1.60	96.00	2.33	94.03
− 15 + 10	1.13	98.53	1.78	97.77	2.91	96.94
− 10 + 5	1.27	99.80	1.95	99.72	2.78	99.72
− 5	0.198	100	0.28	100	0.28	100

Table 4 shows that the cumulative content of fluorapatite − 295 + 53 μm particle size was 72.51% and that of + 20 μm particle size was 96.35%. Dolomite − 295 + 53 μm has a cumulative content of 77.29%, and + 20 μm has a cumulative content of 94.40%. Quartz − 417 + 53 μm had a cumulative content of 79.68%, while quartz + 20 μm had a content of 91.70%. Fluorapatite, dolomite, and quartz had average particle sizes of 117.8 μm, 104.5 μm, and 105.8 μm, respectively. As a result, the three minerals' disseminated grain sizes are close, and they are concentrated in the grain size of − 295 + 53 μm. The reverse flotation technique is the most often used flotation procedure for calcareous and magnesian phosphate rock, mostly for dolomite removal. As a result, when + 53 μm dolomite is entirely dissociated and recycled, the dolomite removal rate can reach 83.17%, significantly improving the grade and recovery rate of P_2_O_5_ in concentrate. It is often assumed that in particle flotation systems, ore particles with complete mineral dissociation between 20 and 100 μm have a greater flotation effect^28,29^. As a result, + 20 μm dolomite is entirely dissociated and recovered, and no particles shorter than 20 μm are formed, resulting in a dolomite removal rate of up to 96%. The disseminated particle size of fluorapatite, dolomite, and quartz in the ore is coarse, and the content of fine particle size is extremely little, indicating that the ore belongs to the type of easy dissociation and may be utilized for one-stage grinding.

The overall particle size distribution trend of the three minerals is the same, as shown in Fig. 8, and the cumulative distribution difference of each particle size of distinct minerals is minor, indicating an equigranular dissemination relationship. The cumulative distribution curves of the three minerals change from steep to sluggish when the particle size is less than 53 μm. As a result, if excessive fine grinding does not aid monomer dissociation, excessive crushing will occur, impairing the flotation effect.

**Figure 9:**
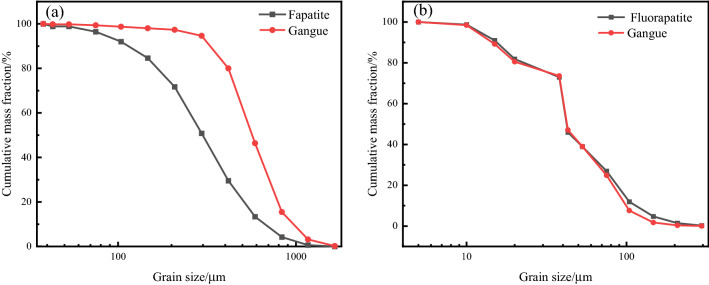
Particle size distribution of fluorapatite and gangue minerals in (**a**) + 250 µm and (**b**) − 250 µm grade ores by XMT.

According to Fig. 9, after the XMT test of the raw ore, the disseminated particle size of fluorapatite is concentrated in − 417 + 104 μm, with a cumulative content of 71.23%, while the disseminated particle size of gangue minerals is concentrated in − 833 + 208 μm, with a cumulative content of 91.45%. The disseminated grain sizes of fluorapatite and gangue minerals are concentrated at − 75 + 43 μm and − 38 + 20 μm for − 250 μm ore particles, with cumulative concentrations of 54% and 57.83%, respectively. Comprehensive study shows that the disseminated particle size of fluorapatite is largely distributed in − 417 μm, of which − 75 + 43 μm and − 38 + 20 μm are greater, and it is also discovered that the mineral content of fluorapatite and gangue in − 20 μm fine particle size is relatively tiny. At the same time, according to Fig. 9, it can be seen that fluorapatite and gangue minerals belong to equal grained dissemination relationship. The grain size of gangue minerals is higher than that of fluorapatite in + 250 μm ore particles, and they are also very dissimilar. The grain size of gangue minerals and fluorapatite in − 250 μm ore particles is small. Therefore, it can also be concluded that the disseminated particle size of the three minerals in the ore is relatively coarse, which belongs to the easily liberated type of ore.

The BPMA and XMT results demonstrate that fluorapatite and gangue minerals are equigranular dissemination. The primary distribution of fluorapatite is − 295 μm according to the results of BPMA-based 2D analysis, and it is − 417 μm according to the results of XMT-based 3D analysis. As a result, the 2D results are smaller than the 3D results. The main reason is that the preparation of samples in the 2D test requires polishing the samples to expose the fluorapatite. However, the size of the exposed surface of fluorapatite determines the size of its embedded particle size. At the same time, there is also stereological deviation in the analysis process, which makes the particle size distribution results finer than the actual^30–32^, and the grinding fineness required for the dissociation of mineral monomers is smaller. The XMT is used for the 3D embedded particle size analysis of fluorapatite. Compared with the particle size analysis based on the 2D plane, XMT is nondestructive testing and the obtained information corrects the stereological deviation, which can avoid the over-grinding of the ore in the grinding process.

### Analysis of combined relationship of main minerals

#### BPMA analysis results

**Figure 10:**
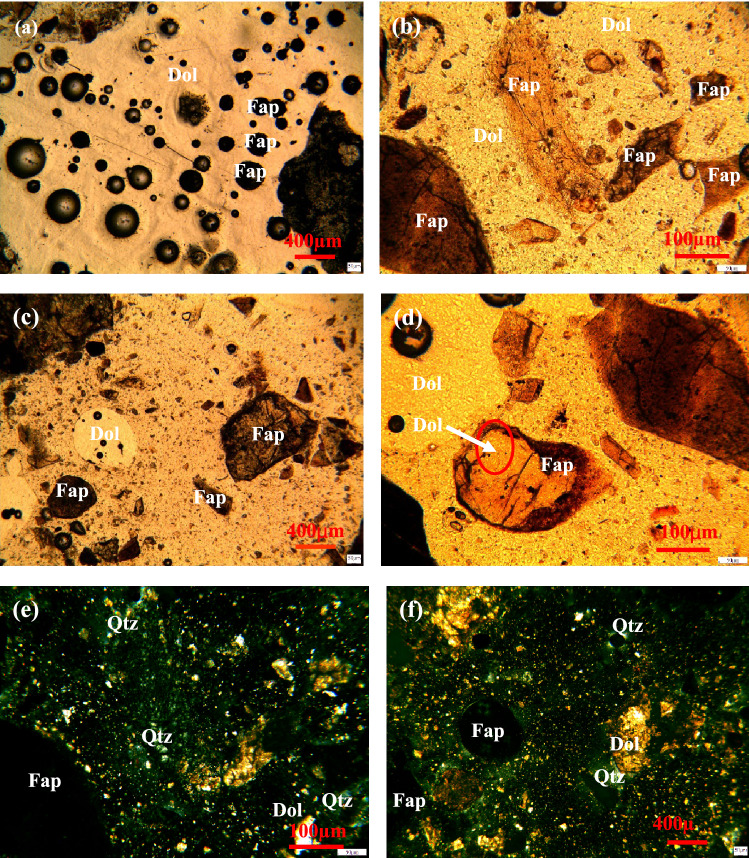
Analysis of shape characteristics of main minerals.

The preliminary analysis results of the three main mineral morphologies in the raw ore and the binding relationship between them are shown in Fig. 10.

**Figure 11:**
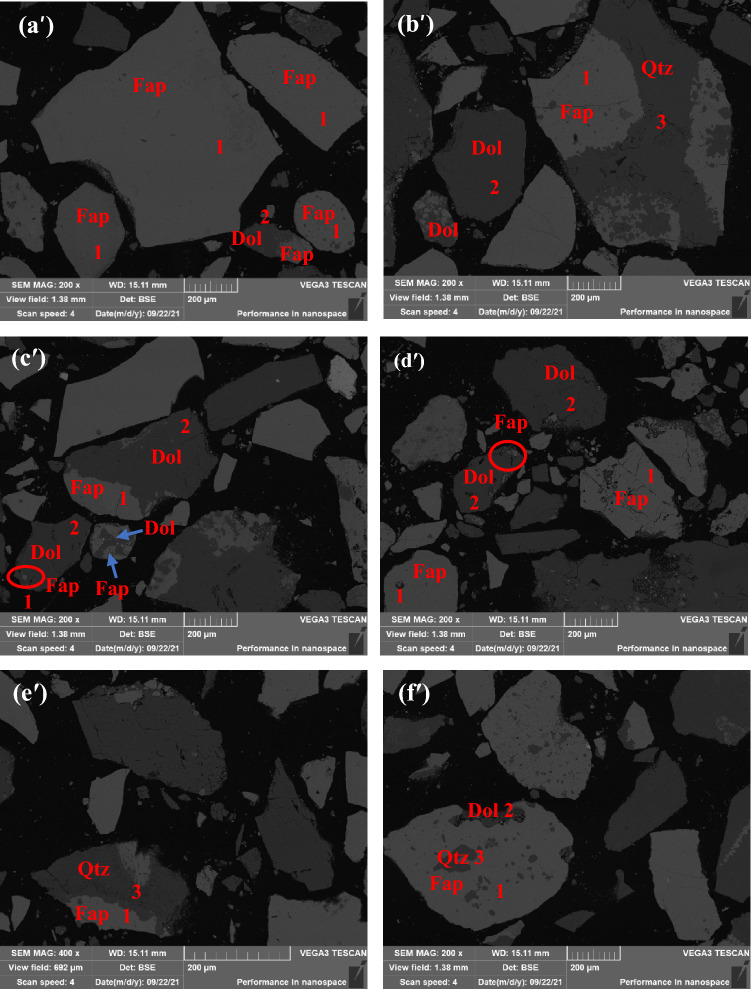
Binding relationship of fluorapatite, dolomite and quartz scanning electron microscopy backscatter.

**Figure 12:**
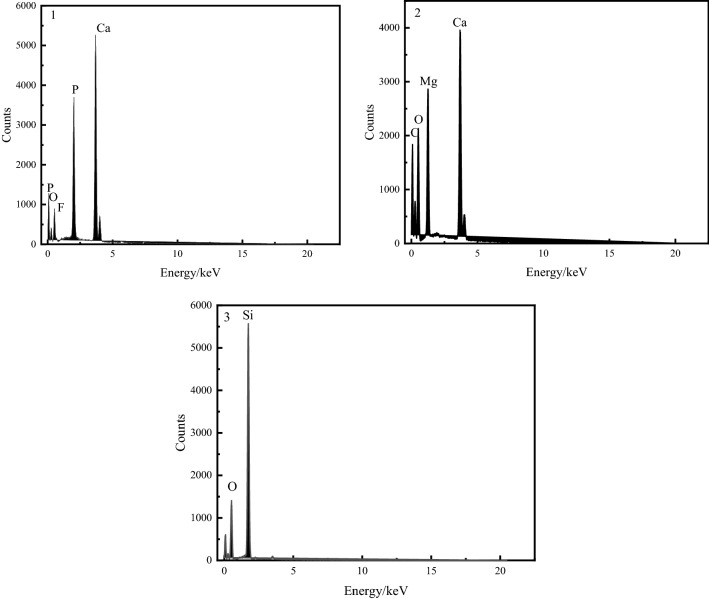
Energy spectrum analysis results of fluorapatite, dolomite and quartz, where 1, 2 and 3 represent fluorapatite, dolomite and quartz in Fig. 11, respectively.

Both fluorapatite and dolomite are semi-transparent minerals, so they are observed and analyzed under single-polarized light. It can be seen that fluorapatite is mainly produced in the form of collophanite. Due to the black deposition and poor transparency, it is black or brown under single-polarized light, and its shape is mainly oolitic, strip and round particles (Fig. 10a,b). Dolomite is yellow-white part (Fig. 10c,d), and its shape is mostly elliptical granular structure. Quartz is a transparent or semi-transparent mineral. It is difficult to distinguish quartz from dolomite under single polarization. Therefore, quartz is observed under orthogonal polarization. Under orthogonal polarization, quartz is gray-white of grade I, showing light blue light (Fig. 10e,f), and its shape is mostly irregular particles and thin layers. The binding relationship of fluorapatite, dolomite and quartz can be further analyzed by scanning electron microscopy. The results are shown in Fig. 11, and the results of energy spectrum analysis of the three minerals are shown in Table 5 and Fig. 12.

**Table 5:** Energy spectrum analysis results of fluorapatite, dolomite and quartz.

Number	C/%	O/%	F/%	Mg/%	Si/%	P/%	Ca/%	Fe/%
1		31.41	6.54			18.43	43.62	
2	6.53	65.10		14.82			13.56	
3		49.24			50.76

The energy spectrum analysis of the three minerals shows that they are fluorapatite, dolomite and quartz in the form of independent minerals. The position of the three types of mineral distribution is further revealed by scanning electron microscopy back scattering image analysis, revealing the combination relationship between the three types of minerals: Fluorine apatite is the most abundant mineral in this ore; the major collection is granular and irregular granular, block structure, not totally liberated, contains a tiny quantity of monomer fluorapatite, and thus primarily from s euhedral and hypautomorphic granular structure. Dolomite is the most abundant gangue mineral, with an euhedral and semi-euhedral granular structure. Dolomite's disseminated grain size is concentrated at + 53–295 µm, with a few grains reaching + 295–589 µm and a few grains less than 20 µm. The coarse-grained dolomite gangue minerals are adjacent to fluorapatite (Fig. 11a′), and some coarse-grained dolomite gangue minerals are interbedded with fluorapatite (Fig. 11c′). However, for fine-grained dolomite gangue minerals, they form an infection relationship with fluorapatite (Fig. 11b′,c′). For fine-grained fluorapatite, they are wrapped in coarse-grained dolomite (Fig. 11c′) and interbedded with coarse-grained dolomite (Fig. 11d′). Quartz gangue minerals are mostly semi-automorphic granular structure, mainly irregular mineral aggregates. Quartz has a maximum disseminated grain size of 417 µm, which is concentrated in the range of + 53–295 µm, and contains extremely few − 20 µm micro-fine particles. It can be seen that coarse-grained quartz runs through fluorapatite in vein (Fig. 11e'), while fine-grained quartz forms complex with fluorapatite.

#### XMT analysis results

**Figure 13:**
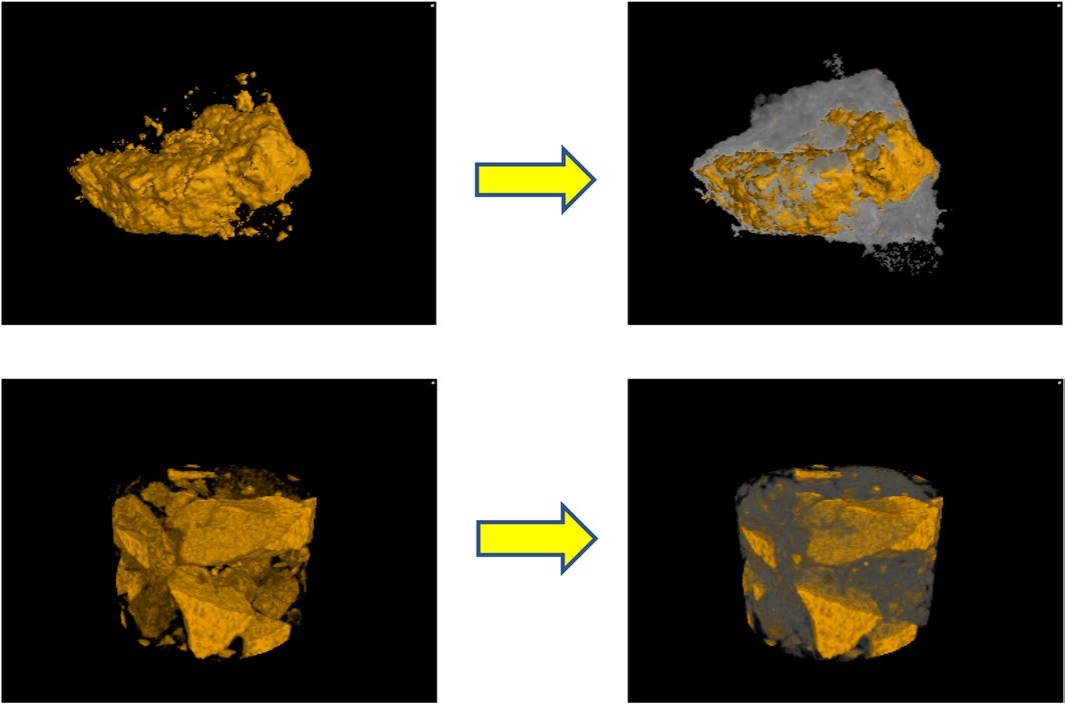
3D mineral dissemination characteristics of fluorapatite and gangue minerals.

The 3D binding relationship between fluorapatite and gangue minerals is shown in Fig. 13. The yellow part represents fluorapatite and the white part represents gangue minerals.

It can be seen from Fig. 13 that the coarse-grained fluoroapatite is mostly dense blocky particles, and there are also debris-like fine-grained fluoroapatite. Fluorapatite and gangue minerals are primarily wrapped, with two types of combination relationships: fully wrapped and incompletely wrapped. Massive fluorapatite with higher particle size has a bigger exposed surface, whereas fine fluorapatite is typically entirely wrapped by gangue minerals. As a result, when the exposure surface of massive fluorapatite is vast but its interior is surrounded with gangue minerals, just the fluorapatite exposure surface is viewed on the 2D plane, which is frequently misinterpreted as a completely dissociated monomer particle. At the same time, it can be seen that the contact relationship between fluorapatite and dolomite and quartz in 2D plane is mainly caused by the uneven exposure surface of irregular coarse fluorapatite on the surface of ore particles or the fine-grained fluorapatite particles are wrapped in gangue minerals in 3D analysis. Therefore, if the raw ore after a period of grinding will still exist a large number of undissociated particles affect the flotation separation effect.

## Further discussion

**Figure 14:**
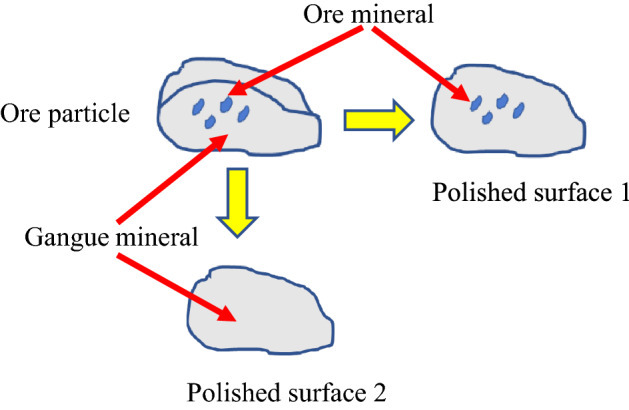
Different degrees of polished surface of ore particles.

BPMA is used to analyze the mineral dissemination characteristics of middle-low grade phosphate ore. The results of mineral distribution, dissemination size distribution and binding relationship analysis are different from those of XMT analysis. The main reason is that the sample preparation process and detection principle of the two methods are different. Although BPMA can accurately distinguish the various mineral phases in the ore, its sample preparation process is cumbersome, and it is necessary to grind the ore particle sample to prepare a polished sheet, which destroys the structural information of the ore sample. Since the binding relationship between the rock and the minerals in the ore is usually wrapped in each other, this feature is also confirmed by XMT analysis of the binding relationship between the useful minerals and gangue minerals in the ore. Therefore, it is difficult to directly observe the 3D spatial size of some small mineral particles using traditional 2D characterization methods. Another reason is that BPMA observes and analyzes the 2D characteristic parameters of the particle section on the polished surface. However, the cross-sectional size and shape of the minerals of the ore particles on the polished surface show great randomness with the relative position between the ore particles and the polished surface. As shown in Fig. 14, if the polished surface is close to the relative position of the ore particles, all mineral information may be obtained, if the relative position between the two is far, the complete information of mineral dissemination characteristics may not be obtained. In this paper, this is also confirmed by the results of two methods of mineral dissemination size analysis. XMT can obtain complete mineral information through non-destructive testing analysis, so compared with the 2D analysis results of BPMA, the mineral dissemination size obtained is coarser.

The main factor affecting the XMT analysis is the discrimination of the attenuation coefficients of different minerals. For low-density non-metallic minerals, their attenuation coefficients are often similar and difficult to distinguish. BPMA is distinguished by the energy spectrum information of different minerals, which can better identify various minerals. Therefore, the advantages of XMT non-destructive analysis in the analysis of mineral dissemination characteristics are unquestionable. It can accurately, clearly and intuitively identify the 3D spatial size, morphology and combined relationship of minerals in ore particles, and provide a better strategy for grinding, but it also needs to be developed and improved. So that XMT can distinguish between similar attenuation coefficient of mineral information, is currently commonly used in combination with a variety of light source energy scanning to distinguish between similar attenuation coefficient of mineral information^33^.

## Conclusion

The mineral dissemination characteristics of − 1 mm medium–low grade calcium magnesium phosphate rock are compared and analyzed using the BPMA and XMT methods in this paper. The results demonstrate that the grain size relationship of the principal minerals is equigranular, and the content of fine-grained minerals is quite low. However, based on the analysis of the 2D mineral dissemination characteristics of BPMA, the results show that the dissemination size of the three minerals is primarily distributed at − 295 µm, with the combination relationship between coarse-grained minerals being mosaic symbiosis and vein penetration, and the fine-grained minerals being complex poikilitic and disseminated, belonging to the ore type of easy liberation and flotation, and the primary grinding. According to the XMT 3D analysis, fluorapatite is primarily dispersed at − 417 µm, while gangue minerals are primarily distributed at − 589 µm. Fluorapatite and gangue minerals are mainly poikilitic. The exposed surface of coarse-grained fluorapatite is relatively large, and most of them are incompletely wrapped with gangue minerals. The exposed surface of fine-grained fluorapatite, on the other hand, is rather tiny, and the majority of them are entirely coated with gangue minerals. If primary grinding process is used, there will still be a large number of contiguous particles affecting the flotation separation effect.

The advantages of the XMT approach include primarily the non-destructive examination of mineral particle size, and the results of mineral particle size analysis are more accurate than those of 2D analysis, and the stereological mistake is corrected, as demonstrated by a comparison of the two methods. Simultaneously, a 3D visualization model of mineral dissemination characteristics is constructed, which can intuitively observe the binding relationship between minerals in the ore. The sample preparation process is simple and does not need to destroy the sample structure, so it can be repeatedly measured. As a result, it can be concluded that XMT can be used as a quick and simple non-destructive testing method in the process mineralogical characterization of medium–low grade calcium magnesium phosphate ore, but for the test analysis of gangue minerals with similar density, 2D analysis methods such as BPMA can be combined, or XMT scanning tests with different intensities of energy can be performed. The data analysis reveals that the XMT test findings are repeatable. As a result, XMT, in addition to other methods such as BPMA, can be used to investigate the grinding products of middle-low grade calcium-magnesium phosphate ore.

As a result, it is possible to infer that XMT can be utilized as a quick and easy non-destructive testing method in the process mineralogy characterization of medium and low-grade calcium magnesium phosphate rock. However, for testing and analyzing gangue minerals of similar density, the BPMA analysis method or an XMT scanning test with varying intensities of energy can be utilized. The data analysis reveals that the XMT test findings are repeatable. As a result, in addition to BPMA, XMT can be used to investigate the grinding products of medium–low grade calcareous-magnesium phosphate rock. XMT technology, in particular, is utilized to measure other critical structural aspects of ore particles, such as spatial location, and these data are used to build the model of mineral dissociation in the crushing process. In particular, XMT technology can be used to quantify other key structural features of ore particles, such as spatial location. These data are used to construct a model of mineral dissociation during crushing.

## Data Availability

All data are available in the main text or the supplementary materials. All the images in the document are from instrument testing and processing analysis, not generated by software. The XMT image data and processing is from the workstation, and the BPMA image is from the instrument comes with SEM, its image processing is mainly automatic analysis of the instrument.
